# Genotype adaptive patterns in spring wheat reveal drought-induced differentiation in root morphology

**DOI:** 10.3389/fpls.2025.1534211

**Published:** 2025-05-21

**Authors:** Nicole Frantová, Lorena Guardia-Velarde, Ivana Jovanović, Martin Weih

**Affiliations:** ^1^ Department of Crop Science, Breeding and Plant Medicine, Mendel University in Brno, Brno, Czechia; ^2^ Department of Crop Production Ecology, Swedish University of Agricultural Sciences, Uppsala, Sweden

**Keywords:** early vigor, root traits, drought, low-temperature, Ppd-D1, Vrn-1, spring wheat

## Abstract

Root morphology of agricultural crops plays a crucial role in crop resilience and early vigor, especially under the increasingly challenging environmental conditions associated with global climate change. This study aims to link root morphology types in six spring wheat genotypes to seedling establishment and stress responses under drought, suboptimal temperature (10°C), and low-temperature (7°C) conditions, providing insights for breeding programs aimed at enhancing early vigor. We quantified early vigor-related root traits (root length and diameter) using a non-invasive phenotyping method, while *PPD-D1* and *VRN-1* genes were analyzed to explore genetic influences on early root development. Results revealed considerable genotypic variation in root length. Stress at both 7°C and 10°C accentuated these differences, with the genotype ‘Dacke’ showing a reduced root length under low-temperature stress. Root diameter responses also varied significantly, with the genotypes ‘Bjarne’ and ‘Dacke’ developing thicker roots than ‘Diskett’ and ‘KWS Alderon’. Genotypic analysis revealed variations in *PPD-D1* alleles, with ‘Bjarne’ carrying the photoperiod-insensitive allele (*Ppd-D1a*), which facilitated early root development as reflected in increased root length, while the other genotypes carried the photoperiod-sensitive allele (*ppd-D1b*). Genotyping of *VRN-A1*, *VRN-B1*, and *VRN-D1* revealed allele combinations associated with a facultative growth habit in all genotypes that were originally classified as spring wheat. This suggests that these genotypes retain some responsiveness to vernalization, despite being used as spring types in practice. These findings highlight the genetic basis of early vigor and root adaptation under stress conditions, providing valuable insights for targeted breeding aimed at improving stress adaptation and resilience in variable climates.

## 1 Introduction

Spring wheat (*Triticum aestivum* L.) serves as a crucial staple crop in regions where harsh winter conditions prevent the successful cultivation of winter wheat, especially when the goal is to produce high protein grain. Traditionally, these regions experience prolonged winter periods that limit the growing season, making spring wheat an essential alternative due to its shorter growing cycle. However, with the advent of climate change, seasonal conditions are becoming increasingly unpredictable, frequently leading to extended winter periods and the emergence of severe drought episodes during critical growth phases (McCabe and Wolock, 2010; Rosner et al., 2018; Walsh et al., 2020). These shifts in weather patterns present significant challenges for farmers, who are now confronted with prolonged exposure to low temperatures during the early growth stages and increased water scarcity during the crop’s development (Li et al., 2014). Consequently, there is an urgent need for the development and cultivation of spring wheat genotypes that exhibit enhanced vigor and resilience to low temperatures at emergence, alongside improved drought tolerance (Zhang et al., 2015; Lan et al., 2022; Vukasovic et al., 2022). These adaptive traits are becoming more critical than ever to ensure the stability and productivity of spring wheat in the face of evolving climatic threats. The vernalization requirements can also be understood as an adaptive trait, differing significantly between winter and spring wheat, and playing a crucial role in their adaptation to varying climate and weather conditions (Yan et al., 2015). Thus, winter wheat typically requires several weeks of exposure to cold temperatures to transition from vegetative growth to flowering, a mechanism that prevents premature flowering in cold climates. Facultative wheat, however, has a much lower vernalization requirement, or sometimes none, allowing it to be planted in either fall or spring, without the need for a prolonged cold period. Spring wheat, on the other hand, does not require vernalization, which allows it to be planted in spring and progress through its growth stages without needing exposure to cold (Hosseini et al., 2021).

In the context of this paper, seed vigor is defined as the early, vigorous growth of roots during the early growing season. Early vigor, often associated with rapid root growth, is a key trait influencing a plant’s ability to access water and nutrients, particularly in suboptimal conditions such as drought. Spring wheat genotypes with higher seed vigor have been shown to exhibit more efficient root proliferation, enhancing their resilience in drought-prone environments (Liu et al., 2021a; Bishopp and Lynch, 2015). Meanwhile, low-vigor seeds represented as the seeds which do not perform well when exposed to stress, were found to have lower content of reserves such as lipid and protein content and increased levels of amino acids, carbohydrates, and phosphorus compounds in the embryo caused by metabolic imbalances or an inefficient use of energy during early growth (Andrade et al., 2020). In cereals, early vigor has been proposed as an essential trait affecting drought tolerance, nutrient uptake, weed competitiveness, and overall yield (Bertholdsson and Kolodinska Brantestam, 2009). In spring wheat, particularly in high-latitude regions with short cropping seasons, early vigor defined as rapid root growth early in the season is crucial (Chawade et al., 2018). By selecting genotypes with favorable early root-growth traits, breeding programs can improve both drought tolerance and nutrient use efficiency, ultimately enhancing yield stability especially in the more extreme weather conditions expected as a consequence of climate change (Liu et al., 2021a). Genotypes with higher early vigor demonstrate faster root extension and proliferation, improving access to water and nutrients in subsoil layers (Palta and Watt, 2009). This is particularly important in resource-limiting environments, where root architectural traits play a significant role in soil resource capture.

The mature wheat root system architecture is closely linked to the angle of seminal root axes at the seedling stage, which influences root depth at later stages (Manschadi et al., 2008). Root length is often pointed out as a key indicator of early vigor, with longer roots and greater diameter generally considered desirable traits (Zeng et al., 2022). However, the optimal root characteristics can vary depending on the specific soil composition and weather conditions of the growing season (Louvieaux et al., 2020). For instance, in compacted soils, plants may benefit from shorter, thicker roots that provide greater structural support. Conversely, in sandy soils, which have lower water retention, plants might develop thinner, longer roots to more effectively forage for water (Popova et al., 2016). Additionally, seedling growth stages are vital for the differentiation of primordia for tillers and spikelets, directly impacting final grain yield (Bertholdsson and Kolodinska Brantestam, 2009; Kirby and Appleyard, 1986).

Studying root growth under field conditions is challenging, especially when involving many cultivars. Alternative methods include studies in pots and root-boxes, though these also limit the number of cultivars that can be handled (Virlet et al., 2017; Rutkoski et al., 2016). The unpredictability of the natural environment means that drought stress can threaten wheat at any growth stage throughout its life cycle (Senapati et al., 2019). While late drought is often associated with more significant yield losses, in Scandinavian countries like Denmark and Sweden, spring wheat often faces drought during late spring, particularly in the crucial early stages of crop development, when the roots are still underdeveloped (Lan et al., 2022; Van Ginkel et al., 1998). However, genetic factors also play a crucial role in determining early vigor, particularly through specific genes and their allelic variations, which can impact various aspects of plant development, including root morphology. Among genetic factors of interest, the alleles of *PPD-D1* and *VRN-1* are noteworthy due to their potential influence on root traits, particularly under stress conditions such as drought (Voss-Fels et al., 2017; Makhoul et al., 2024).

In hexaploid bread wheat (*Triticum aestivum*, AABBDD), the *VRN-1* and *PPD-1* genes exist as three homoeologous copies, located on the A, B, and D genomes (*VRN-A1, VRN-B1, VRN-D1* and *PPD-A1, PPD-B1, PPD-D1*). The *VRN-1* gene plays a central role in determining the vernalization requirement, and its allelic variation (dominant (spring) vs. recessive (winter)) defines whether a wheat genotype is classified as winter, facultative, or spring. Spring wheat genotypes typically carry dominant alleles at all three *VRN-1* loci, allowing flowering without prolonged cold exposure. However, many cultivars described as “spring” may harbor a mixture of spring and recessive winter alleles, especially at the *VRN-B1* or *VRN-D1* loci, in which case they are considered facultative (Fu et al., 2005; Yan et al., 2015). *VRN-1* is a key transcription factor involved in the vernalization pathway, which regulates the transition from vegetative to reproductive growth in response to prolonged cold exposure (Deng et al., 2015).

Regarding photoperiod sensitivity, bread wheat also possesses three *PPD-1* homoeologs. Among them, *PPD-D1* is the most influential in regulating flowering time in response to day length (Beales et al., 2007). While other photoperiod genes such as *PPD-A1*, *PPD-B1*, and *PPD-2* exist, *PPD-D1* has shown the strongest phenotypic effect on heading time, particularly in European and Asian germplasm, which justified its selection for this study. *The PPD-D1* gene encodes a pseudo-response regulator that is primarily known for its role in controlling photoperiod sensitivity, thus affecting the timing of flowering and other developmental processes in wheat (Gomez et al., 2014).

Although the mentioned genes are traditionally studied in the context of flowering time and developmental timing, emerging research suggests they may also influence root development. Roots are vital organs for water and nutrient uptake, especially under drought conditions where efficient root systems can enhance plant survival and productivity (Comas et al., 2013). Based on these insights, these extensively studied wheat genes were chosen to investigate their potential impact on root architecture in this study. While the direct relationship between *PPD-D1* and *VRN- 1* and root morphology, such as root length and diameter has not been conclusively established, there is growing evidence that these genes may exert indirect effects on root development. For instance, modifications in the expression of *PPD-D1* can lead to changes in the timing of developmental stages, which may, in turn, affect the early growth rate and vigor of roots (Foulkes et al., 2004). Similarly, *VRN-1*, through its role in regulating developmental transitions, could influence the allocation of resources during critical growth periods, potentially impacting root architecture and resilience under stress (Voss-Fels et al., 2017).

This study aimed at linking different root morphology types of spring wheat grown under osmotic stress to seedling establishment and plant responses to drought, suboptimal and low-temperature conditions, which will provide insights to be used in future plant breeding. In order to connect the phenotypic pattern with some genetic background, basic genotyping analyses were performed to offer genetic insights into these genotypes, with the analysis of *PPD-D1* and *VRN-1* genes adding valuable context for interpreting the genetic influences on root development. By examining how roots adapt to abiotic stresses, particularly drought and low-temperature conditions at sowing, this study addresses the growing interest in early vigor-related root traits. These traits are especially crucial for spring crops in northern latitudes, where long photoperiods and short growing seasons require rapid and resilient early growth. We expected the genotypes to vary in their response to drought, suboptimal and low-temperature conditions by alterations in root length and diameter, to optimize resource acquisition under different stress conditions.

We explored the following hypotheses: (H1) There is significant genetic variation in root growth responses to osmotic stress at low temperatures, with some genotypes showing a greater ability to maintain or extend root length under low-temperature and drought stress. (H2) Prolonged exposure to combined stress conditions (low temperatures and drought) leads to significant variation in root diameter among genotypes. (H3) The correlation between root length and diameter varies across genotypes, suggesting diverse patterns in adjusting root morphology to maintain or improve resource acquisition under stress. These differences in phenotypic pattern are expected to be reflected in the allele presence or absence for particular genes involved in shaping root traits (e.g., *PPD-D1* and *VRN-1*).

## 2 Materials and methods

### 2.1 Genotype selection

In this study, six genotypes represented by commercially available varieties of spring wheat (*Triticum aestivum* L.) were selected for evaluation, chosen specifically to ensure genetic diversity and minimal genetic relatedness. The selected genotypes include ‘Bjarne’, ‘Rohan’, ‘KWS Alderon’, ‘Diskett’, ‘Quarna’, and ‘Dacke’ (**Table 1**). Under high-latitude conditions, previous studies in Sweden and Norway have identified ‘Bjarne’ as an early-heading genotype (Uhlen et al., 2015). These genotypes were purposefully chosen as they do not share significant common ancestry in their parental lines, thereby reducing the likelihood of genetic overlap and ensuring a broad representation of the genetic variability within the species. As the seeds were harvested from field conditions, (**Supplementary Figure 1**) provides information about the weather and precipitation conditions that affected the growth of maternal plants and the ripening of grains.

**Table 1:** Origin, pedigree, and habit of the spring wheat genotypes used in this study.

Genotype	Origin	Pedigree	Habit
BJARNE	Norway	SV-B-87293/BASTIAN	spring
DACKE	Sweden	P-18/17269//19151	spring
DISKETT	Sweden	VINJET/4499979//BALDUS/SW-33154	spring
ROHAN	Sweden	Not available	spring
QUARNA	Switzerland	Not available	spring
KWS ALDERON	Germany	CPBT-W-110/BELVOIR//TYBALT	spring

### 2.2 Genomic DNA extraction and molecular marker analysis

DNA was extracted from young leaves of wheat seedlings grown at room temperature using the DNeasy Plant Mini Kit (Qiagen, Germany) following the manufacturer’s instructions. PCR markers were used to detect allelic variants at the *PPD-D1* and *VRN-1 loci*, as described by Beales et al. (2007); Fu et al. (2005), and Yan et al. (2015). PCR conditions were optimized using standard Taq polymerase protocols, and amplification products were visualized on agarose gels. Further details on primer sequences, reaction conditions, expected product sizes, and reference genotypes are available in **Supplementary Material**. Additionally, for *VRN-A1* allele identification, two commercially available European wheat genotypes were used as references: Bohemia (winter wheat with a strong vernalization requirement of approximately six weeks) and Tybalt (a facultative type that senses vernalization but does not require it).

### 2.3 Early vigor assessment

The first step involved sorting the healthy-looking grains, followed by immersing them in 3% sodium hypochlorite for 10 minutes and rinsing them three times with distilled water. The evaluation of early vigor in spring wheat was conducted under drought conditions and suboptimal temperatures. Drought conditions were simulated using a polyethylene glycol (PEG 6000) solution to achieve a water potential of - 0.5 MPa, as described by Michel and Kaufmann (1973). Seed germination was assessed at two temperatures: 10°C, which we considered suboptimal for germination and 7°C, chosen to evaluate cold tolerance as it is closer to the minimum temperature required for growth. Although 4°C was initially considered, preliminary observations confirmed that all physiological processes ceased at this temperature in combination with an osmotic potential of - 0.5 MPa, leading to stunted growth. Therefore, 7°C was used as a low-temperature treatment, being closer to the minimum temperature threshold, to better assess the effects of cold stress. A control group was subjected to a standard germination test under optimal conditions following the ISTA standard method. Accordingly, this experiment included three treatments: optimal temperature (20°C) without osmotic-stress (‘control’); suboptimal temperature (10°C) with osmotic-stress; and closer to minimum temperature for growing conditions (7°C, simulating cold stress) with osmotic-stress.

Six seeds of each wheat genotype were placed in separate 90 mm diameter sterile Petri dishes. Each dish contained 8 ml of the PEG 6000 solution, except for control which contained distilled water. Petri dishes were sealed in plastic bags to prevent evaporation of the PEG solution. The sealed dishes were then placed in a climate-controlled chamber set at 10°C in complete darkness. The experiment was designed with three repetitions and four replications of each variant (control, drought and cold conditions). Germination rates and vigor were assessed after 7 and 14 days. Early vigor was assessed by measuring root lengths and diameter using a scanner. The germinated seeds from the Petri dishes were scanned and analyzed using WinRHIZO (Régent Instruments Inc., Quebec, Canada), version 2020 Arabidopsis. The analysis was conducted on six seeds for each repetition within each replication. Each scanned image was thoroughly examined, and using the exclusion tool, any elements that did not belong to the roots were removed. To assess early vigor under drought conditions in Petri dishes, measurements were taken for root length (cm) and average root diameter (mm) after seven and fourteen days.

### 2.4 Statistical analysis

Non-parametric testing was conducted using the Kruskal-Wallis test with *post-hoc* analysis performed using the Dwass-Steel-Critchlow-Fligner (DSCF) test. These tests were chosen based on the results of the Levene’s test for homogeneity of variances (*p* < 0.001) and the Shapiro-Wilk test for normality (*p* < 0.001), which indicated that the assumptions required for parametric ANOVA were not met.

Spearman’s rank correlation coefficient analysis was performed to study the relationship between root length and diameter under different conditions. This analysis was conducted separately for each treatment and genotype to account for the possibility that each environment could influence the behaviour of the tested genotypes differently.

For graphical representation, box plots and standard Principal Component Analysis (PCA) were used. For PCA, the data were standardized using z-score normalization to ensure a mean of 0 and a standard deviation of 1, which is essential given that root length and diameter are measured on different scales (cm and mm). After the data were standardized, a covariance matrix was calculated to show the relationships between the variables. This matrix was then decomposed into eigenvalues and eigenvectors. The eigenvalues indicate how much variation each principal component captures, while the eigenvectors define the direction of these components. Finally, the standardized data were projected onto the eigenvectors to calculate the principal components.

The graphical representations were created using Python, specifically the Matplotlib library for box plots and the Scikit-learn library for PCA. Statistical analyses were conducted using Python and Jamovi.

The size of the dataset was n = 360, when testing individual treatments n = 72 and for each genotype n = 18. All statistical test results were deemed statistically significant if the *p*-value was less than 0.05.

All statistical results, including detailed outputs from the extensive analyses, are provided in the (**Supplementary Tables 1–6**) due to the large volume of tests conducted.

## 3 Results

### 3.1 Genotype and stress treatment affected root diameter and length relationships

**Figure 1:**
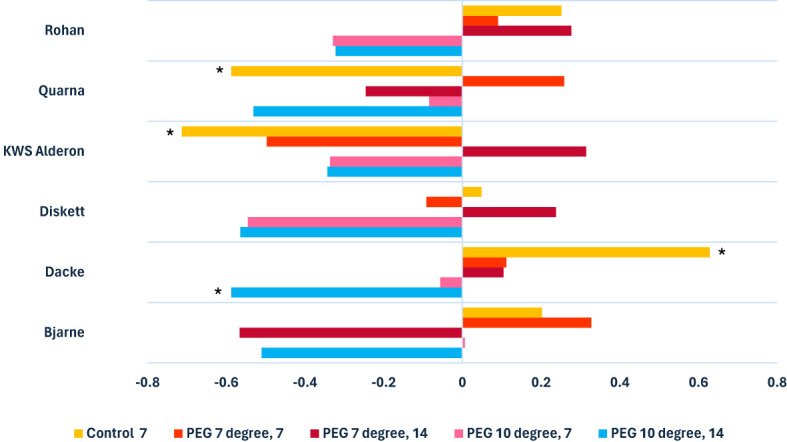
Clustered bar chart illustrating Spearman’s rank correlation coefficients, with * indicating statistically significant positive and negative correlations between root lengths and diameters of six spring wheat genotypes grown in Petri dishes and assessed 7 and 14 days after seed germination.

In general, the strength and direction of the correlation between root length and diameter differed significantly between genotypes, depending on the treatment condition applied (**Figure 1**). Under control conditions, a clear positive or negative correlation between root length and diameter was observed. For example, ‘Dacke’ exhibited a positive correlation between the two traits, whilst ‘KWS Alderon’ and ‘Quarna’ showed significantly negative correlations. However, under low-temperature and suboptimal conditions (7°C and 10°C), these genotype-specific patterns weakened or disappeared. In particular, ‘Dacke’ exhibited no significant correlation under stress, indicating a shift in root trait behavior from the control to the stress treatment. Similarly, the negative correlations observed in ‘KWS Alderon’ and ‘Quarna’ under control were not seen under the stress treatments. Root diameter responses were particularly sensitive to combined stress conditions (**Figure 1**). For instance, in the 10°C PEG treatments after 14 days, a moderate negative correlation emerged between genotypes, with some, such as ‘Dacke’, exhibiting significantly thinner roots as root length increased. Genotypic responses varied significantly across treatments. Under control conditions, ‘Dacke’ exhibited a positive correlation between root length and diameter, while ‘KWS Alderon’ showed a strong negative correlation. However, under drought and temperature stress, specifically at suboptimal (10°C) and low-temperature (7°C) conditions, most genotypes showed weaker correlations. Some, such as ‘Diskett’, showed no significant correlation in the PEG treatment at 10°C for 7 days.

### 3.2 Osmotic and temperature stress amplifies genotypic differences in root length and diameter

**Figure 2:**
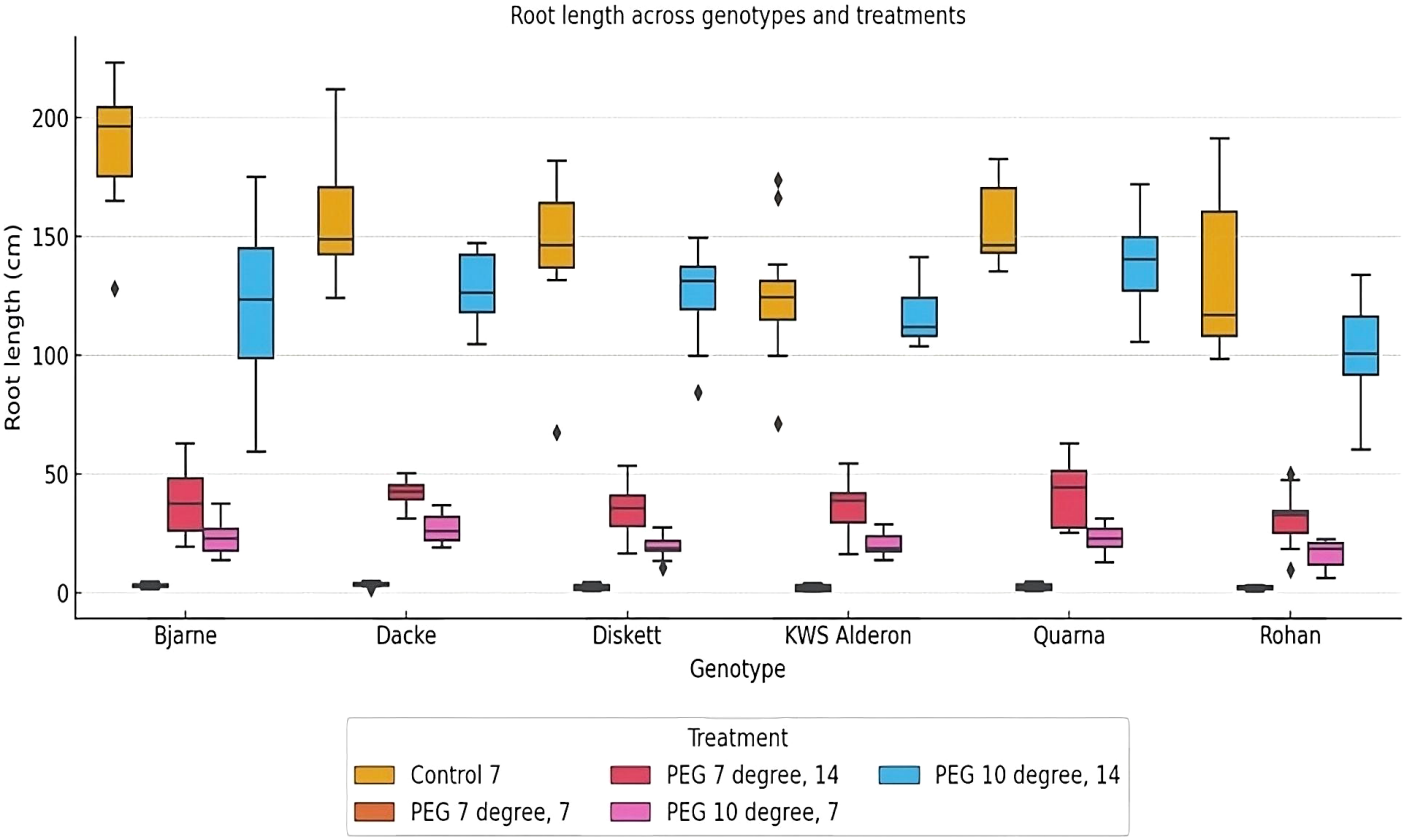
Box plot displaying root length (cm) variations between six spring wheat genotypes grown in Petri dishes for 7 or 14 days under control and osmotic stress treatments at 7°C and 10°C. The boxes represent the interquartile range (IQR), with the line inside each box indicating the median root length. The whiskers extend to 1.5 times the IQR, and outliers are shown as individual points. Significant differences in root length (*p* < 0.05) are observed among genotypes and treatments. .

The analysis of root length across different genotypes and treatments revealed significant genotypic variation. Under control conditions, ‘Bjarne’ exhibited significantly longer roots compared to ‘Diskett’, ‘KWS Alderon’ and ‘Quarna’ (**Figure 2**). When exposed to osmotic stress at both 7°C and 10°C, the variation in root length among genotypes became more distinct. In the 7°C, 7-day PEG treatment, the genotype ‘Dacke’ exhibited shorter roots compared to ‘Diskett’ and ‘KWS Alderon’, indicating a reduced ability to maintain root growth under stress. For better resolution, the values for the PEG 7°C, 7-day treatment, which showed notably low root lengths, were separated into an additional chart included in the (**Supplementary Figure 2**) to ensure clearer visualization of the differences among genotypes. After 14 days of PEG treatment at 7°C, no significant differences in root length were observed between genotypes. At 10°C, significant variation in root length was found between the genotypes, with ‘Dacke’ showing longer roots compared to ‘Rohan’ and ‘Diskett’ (**Figure 2**).

**Figure 3:**
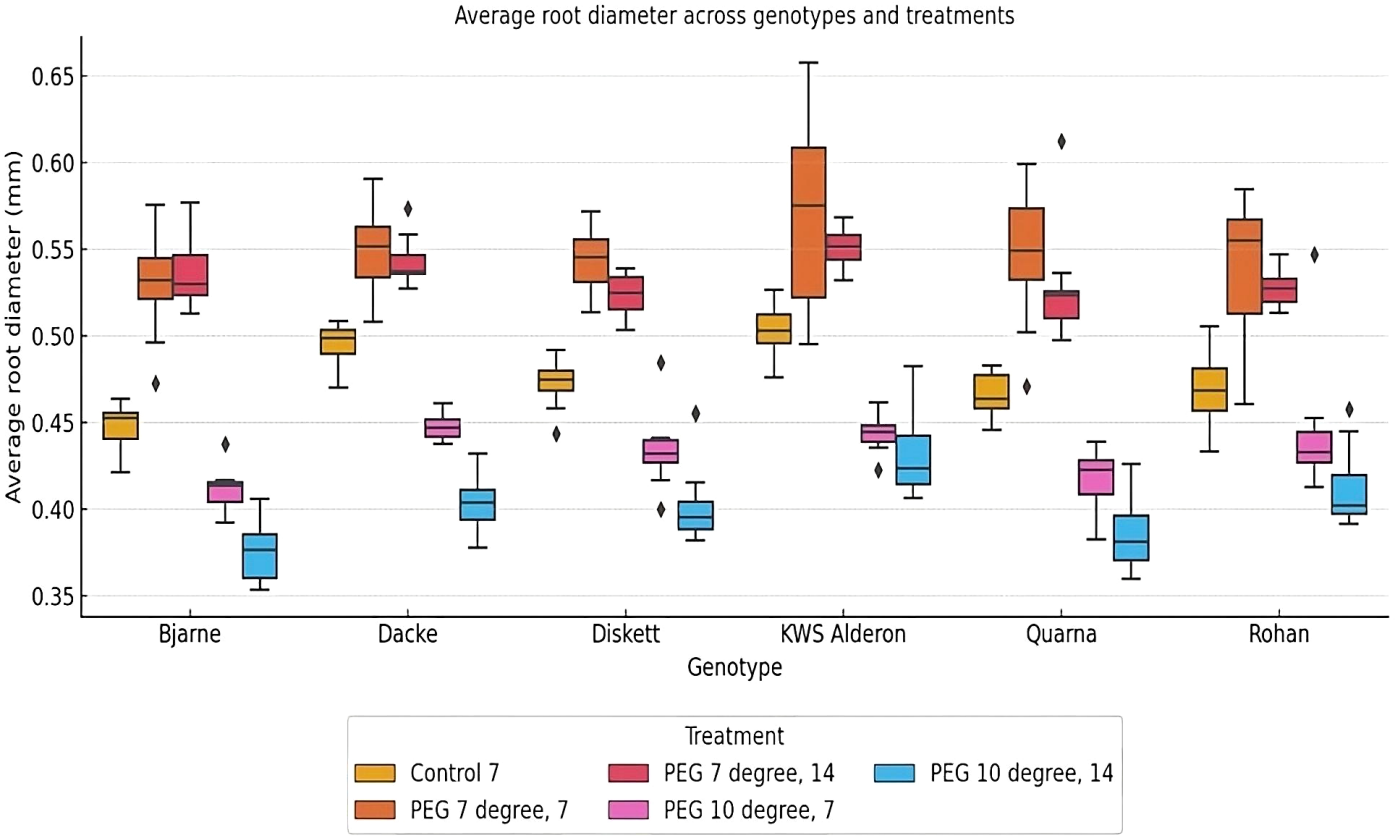
Box plot showing average root diameter (mm) variations between six spring wheat genotypes grown in Petri dishes for 7 or 14 days under control and osmotic stress treatments at 7°C and 10°C. The boxes represent the interquartile range (IQR), with the median root diameter indicated by the line inside each box. Whiskers extend to 1.5 times the IQR, with outliers represented as individual points. Significant differences in root diameter (*p* < 0.05) are observed between genotypes and treatments.

There was significant genotypic variation in root diameter responses to the stress conditions applied here. Thus, under control conditions, the genotypes ‘Bjarne’ and ‘Dacke’ exhibited thicker roots compared to ‘Diskett’ and ‘KWS Alderon’ (**Figure 3**). When subjected to osmotic stress (PEG at 7°C and 10°C), the genotypic variation in root diameter increased, indicating that different genotypes responded differently to stress (**Figure 3**). After 14 days of low-temperature (7°C) osmotic stress, significant differences in root diameter were observed between ‘Dacke’ and ‘Quarna’. At 10°C, similar patterns emerged, with ‘Bjarne’ and ‘Dacke’ consistently showing larger root diameters than ‘Diskett’ and ‘Quarna’.

### 3.3 PCA exploration of root characteristics

**Figure 4:**
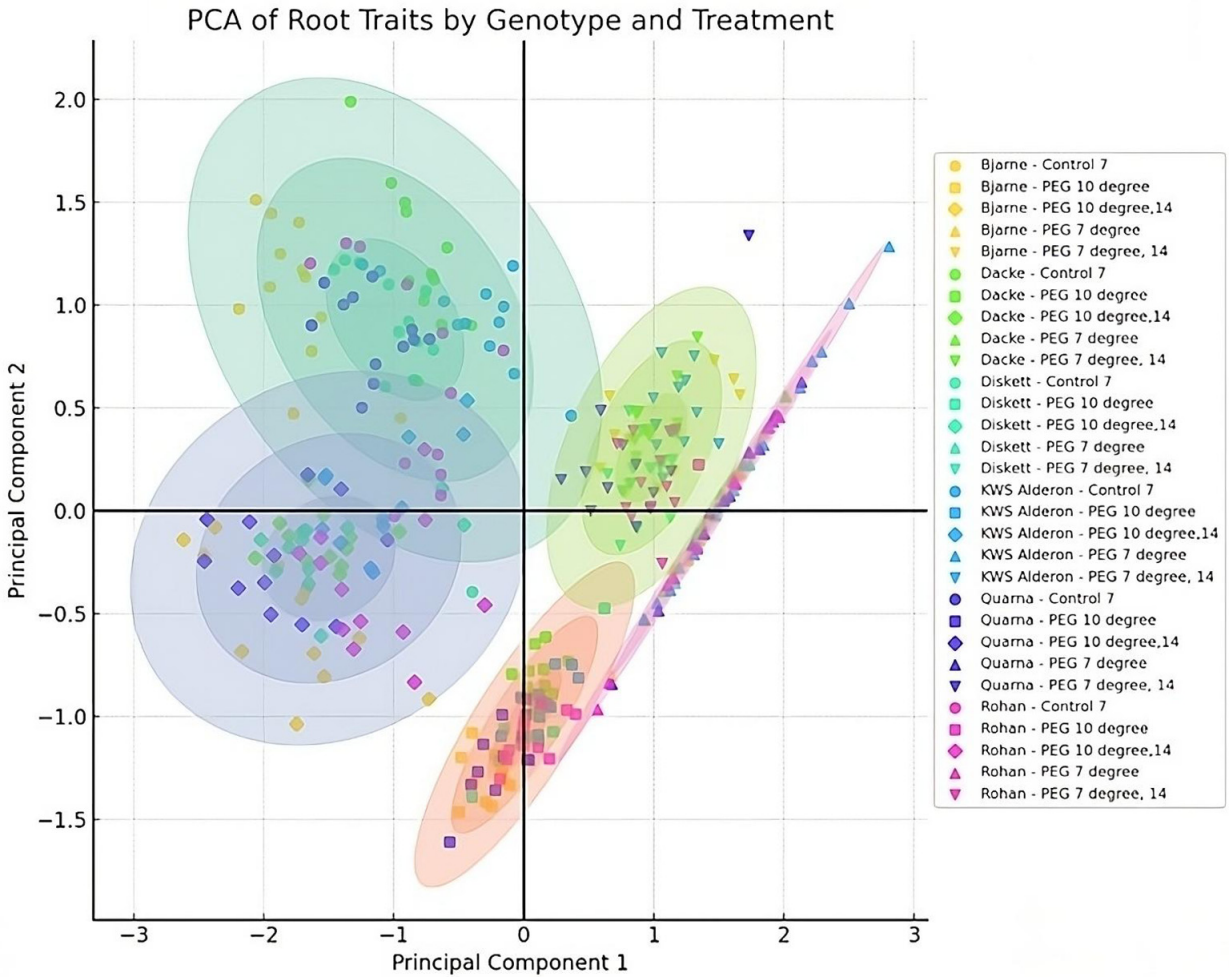
Principal Component Analysis (PCA) showing genotype responses of six spring wheat genotypes grown in Petri dishes for 7 or 14 days under control and osmotic stress treatments at 7°C and 10°C. Principal Component 1 (PC1) explains 73.53% of the variance, while Principal Component 2 (PC2) accounts for 26.47%. Clustering patterns indicate genotypic responses to control, temperature, and osmotic stress treatments.

The correlations between root length and diameter under varying conditions revealed how the investigated genotypes responded biologically to both temperature and osmotic stress. These patterns are further illustrated by the principal component analysis (PCA) results (**Figure 4**), which reflect shifts in root development under different treatments. Under control conditions, the majority of genotypes clustered in Quadrant II, displaying a broad dispersion. When osmotic stress was introduced using PEG at 10°C, a noticeable shift occurred after 7 days, with genotypes clustering more closely in Quadrants III and IV. This convergence implies a temporary homogenization in root growth responses. After 14 days under the same conditions, the data points became more dispersed within Quadrant III, suggesting that genotypes began to diverge in their responses. At 7°C, the responses were notably different. After 7 days of combined low temperature and osmotic stress, data points followed a trajectory from Quadrant IV to Quadrant I, indicating a shift in root characteristics. After 14 days, genotypes formed a tighter cluster in Quadrant I, indicating that their root growth responses became more aligned, suggesting a stabilization of the adaptive response under sustained cold and osmotic stress.

### 3.4 Photoperiod insensitivity in ‘Bjarne’ linked to *PPD-D1a*

**Figure 5:**
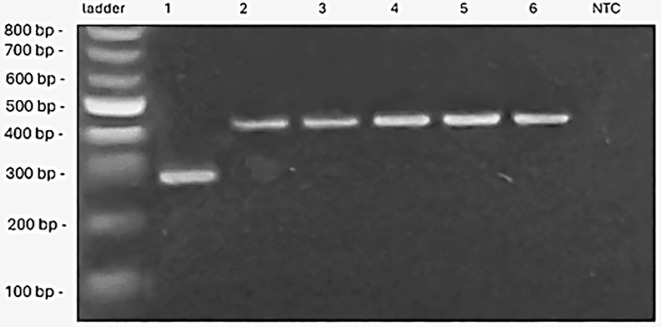
The PCR products of *PPD-D1* alleles identified for six spring wheat genotypes. A dominant allele *PPD-D1a* (288 bp, insensitive to photoperiod), recessive allele *ppd-D1b* [414 bp, sensitive to photoperiod), in the figure from left to right (ladder 100 bp, genotypes ‘Bjarne’, ‘Dacke’, ‘Diskett’, ‘Rohan’, ‘Quarna’, ‘KWS Alderon’, NTC (no-template control)].

Photoperiod sensitivity, regulated by the *PPD-D1* gene, was analyzed across different genotypes. The genotype ‘Bjarne’ exhibited the insensitive allele (*PPD-D1a*), which allows earlier flowering irrespective of the photoperiod. In contrast, ‘KWS Alderon’ and the other genotypes investigated here exhibited the sensitive allele (*ppd-D1b*), indicating a normal response to photoperiod and flowering timing dependent on day length (**Figure 5**).

### 3.5 Distinct vernalization requirements in ‘Dacke’ and ‘Quarna’

**Figure 6:**
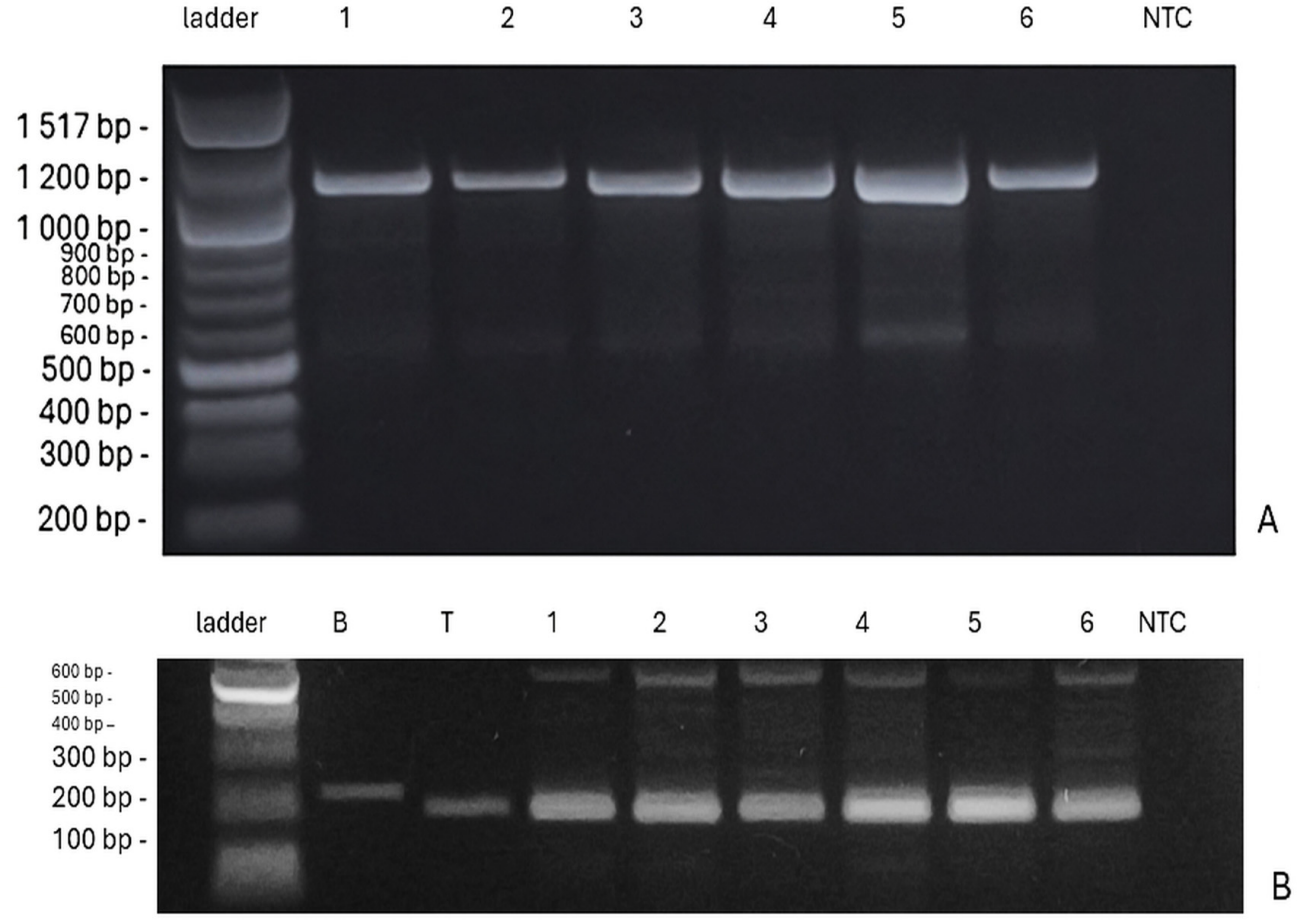
**(A)** PCR amplification of *vrn-A1* (intron 1) showing a 1,068 bp product specific to the winter allele in six wheat genotypes. Lanes from left to right: 100 bp ladder, ‘Bjarne’, ‘Dacke’, ‘Diskett’, ‘Rohan’, ‘Quarna’, ‘KWS Alderon’, and NTC (no-template control). **(B)** Restriction digestion of *VRN-A1* exon 4 fragment (~221 bp) to distinguish between spring and winter alleles based on SNP polymorphism. The winter wheat genotype Bohemia **(B)** shows an undigested 221 bp product, characteristic of the recessive *vrn-A1b* allele (strong vernalization requirement). The facultative genotype Tybalt (T) shows a digested product of 199 bp, corresponding to the dominant *Vrn-A1a* allele. All tested genotypes displayed a fragment pattern similar to Tybalt, supporting their classification as facultative. Faint or additional bands (e.g., ~600 bp) may result from incomplete digestion or nonspecific amplification.

**Figure 7:**
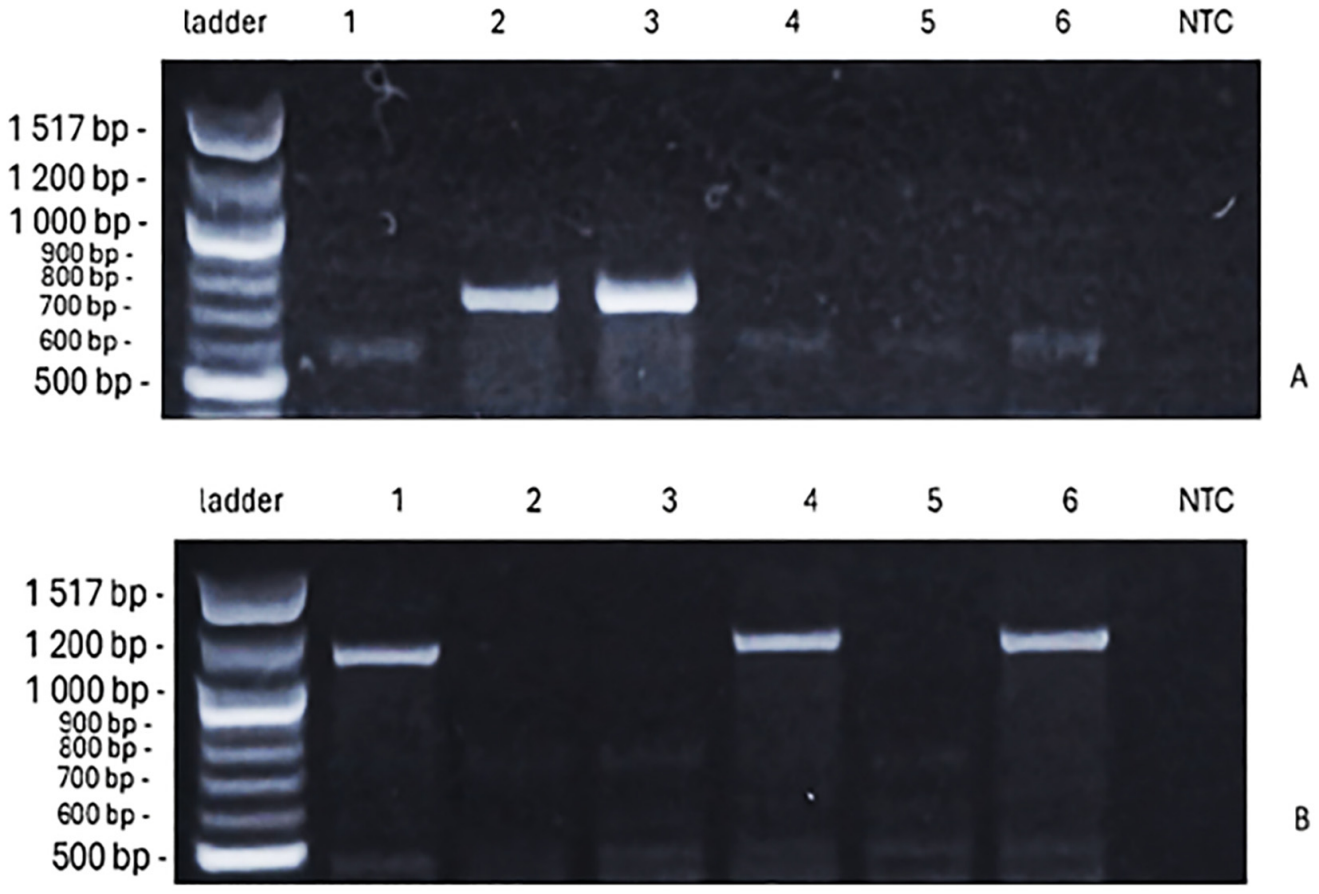
The PCR products of *VRN-B1* alleles [709 bp, spring, **(A)**; 1–149 bp, winter, **(B)**] identified for six spring wheat genotypes, in the figures from left to right [ladder 100 bp, genotypes ‘Bjarne’, ‘Dacke’, ‘Diskett’, ‘Rohan’, ‘Quarna’, ‘KWS Alderon’, NTC (no-template control)].

The presence of *VRN-1* genes, which determine the vernalization requirement, showed variation across the genotypes (**Figure 6A**). The genotypes ‘Bjarne’, ‘Rohan’, and ‘KWS Alderon’ exhibited winter alleles (*vrn-A1/vrn-B1*), while ‘Dacke’ and ‘Diskett’ displayed a combination of spring and winter alleles. Specifically, ‘Dacke’ carried the spring allele *VRN-B1*, indicating a reduced vernalization requirement, and ‘Diskett’ also possessed the *VRN-B1* spring allele (**Figures 7A, B**). Notably, ‘Quarna’ had a mutation in the *VRN-B1* gene, suggesting a divergent vernalization response compared to the other genotypes.

To further refine the classification of growth habit, a restriction digest of exon 4 of *VRN-A1* was performed (**Figure 6B**). In this assay, ‘Bohemia’, a winter wheat with strong vernalization requirement, showed a 221 bp fragment typical of the *vrn-A1a* allele (around 6 weeks vernalization requirements), while ‘Tybalt’, a facultative wheat with minimal vernalization response, exhibited a 199 bp fragment corresponding to the *Vrn-A1b* allele. All tested genotypes produced a band of similar size to ‘Tybalt’, supporting their classification as facultative wheat. Additionally, the samples showed an extra band around 600 bp, which may indicate incomplete digestion, nonspecific amplification, or heteroduplex formation, though the primary 199 bp band remains the definitive marker for allele classification.

**Figure 8:**
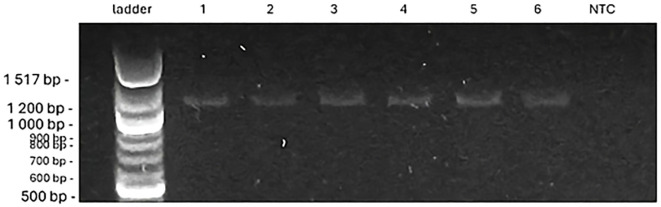
The PCR products of *vrn-D1* allele, which are of a different size from the designed (997 bp, winter), as identified for six spring wheat genotypes. In the figures from left to right [ladder 100 bp, genotypes ‘Bjarne’, ‘Dacke’, ‘Diskett’, ‘Rohan’, ‘Quarna’, ‘KWS Alderon’, NTC (no-template control)].

For *VRN-D1*, the PCR products further contributed to understanding genotype responses. No amplification products were observed for the spring allele *VRN-D1*. However, products for the winter allele *vrn-D1* were present but differed in size (**Figure 8**), potentially reflecting allele variation or partial sequence divergence. Specifically, the results show that ‘Rohan’ and ‘KWS Alderon’ carry classical winter alleles; whilst ‘Dacke’ and ‘Diskett’ carry VRN-B1 spring alleles and exon 4 SNPs in VRN-A1, indicating a facultative growth habit.

## 4 Discussion

The investigation and identification of adaptive crop traits that confer enhanced vigor and resilience to low temperatures especially at seedling emergence, along with improved drought tolerance, is today a high priority in breeding research (Zhang et al., 2015; Lan et al., 2022; Vukasovic et al., 2022). This study primarily examined early vigor root-growth traits in six spring wheat genotypes under osmotic stress, suboptimal, and low temperature conditions, with a specific focus on plant responses to drought. By linking phenotypic patterns to the presence or absence of particular genes (*PPD-D1* and *VRN-1*), the study provides valuable insights that may aid future breeding efforts to enhance stress resilience. Among the wheat genotypes examined, substantial variation in root responses emerged under low temperatures. For example, ‘Dacke’ displayed shorter roots compared to ‘Diskett’ and ‘KWS Alderon’ under 7°C osmotic stress, highlighting its limited capacity for root growth under drought and low temperature conditions. In contrast, ‘Bjarne’ consistently developed the longest roots under control conditions. This indicates diverse baseline root growth characteristics, with some genotypes favoring longer roots and others thicker roots in the absence of drought and/or cold stress. Such variability suggests inherent differences in root architecture and growth regulation. The results also highlight that root length variation can be strongly influenced by stress duration and intensity, with certain genotypes showing greater resilience by maintaining longer roots. This suggests that distinct root growth responses may offer competitive advantages under challenging conditions. Genetic analysis further supported these phenotypic variations, particularly in the photoperiod sensitivity and vernalization response genes. ‘Bjarne’ carrying the photoperiod-insensitive allele *PPD-D1a*, exhibited early flowering regardless of day length, while other genotypes, such as ‘KWS Alderon’ displayed the photoperiod-sensitive allele *ppd-D1b*, aligning with delayed flowering. Additionally, ‘Dacke’ and ‘Diskett’ exhibited spring alleles in *Vrn-B1*, reducing their vernalization requirement, while ‘Quarna’ showed a unique mutation in *Vrn-B1*, potentially influencing its distinct vernalization response.

These genotypes are widely cultivated in Sweden, and were here selected for their relevance to local agriculture. The pedigree analysis (**Table 1**) revealed distinct genetic backgrounds for the studied genotypes, and these were also classified based on their breeding goals (Guardia-Velarde et al., 2023). This classification provides a breeding context that may help further explain and discuss the obtained results. According to Guardia-Velarde et al. (2023), the here investigated wheat genotypes were grouped as follows: high-yield genotype (‘KWS Alderon’), high-protein-content genotypes (‘Quarna’, ‘Dacke’, ‘Bjarne’), and intermediate genotypes (‘Diskett’, ‘Rohan’). The different breeding goals could be reflected in differential rooting responses in extreme environments, as thicker roots seem to be associated with high-yield genotypes and thinner roots are more typical for high-protein genotypes. Differential root characteristics have in turn been shown to be associated with different nutrient use characteristics of the plant material (Liu et al., 2022b), as is further discussed below.

### 4.1 Evidence for adaptive root elongation patterns in wheat genotypes under low temperature stress

In this study, the genotypic differences in root length growth observed under stress conditions revealed diverse adaptive patterns, supporting our first hypothesis (H1) that osmotic stress induces significant genotypic variation in root growth. Under optimal (control) conditions, ‘Bjarne’ exhibited significantly longer roots compared to ‘Diskett’, ‘KWS Alderon’, and ‘Quarna’. This suggests that ‘Bjarne’ has a greater capacity for rapid root elongation, especially in non-stressful environments, reflecting a great potential for efficient resource acquisition under optimal conditions. When exposed to osmotic stress, the variation in root length across the investigated genotypes increased, particularly for the 7-day PEG treatment at 7°C. This temperature, coupled with osmotic pressure, provided a challenging environment for these plants, where the genotypic differences became evident. Under these challenging conditions, ‘Dacke’ exhibited significantly reduced root elongation compared to ‘Diskett’ and ‘KWS Alderon’, emphasizing a differential capacity of these wheat genotypes to maintain root growth under stress (Awad et al., 2018). The sharp decline in root elongation observed in ‘Dacke’ when compared to the less stressful conditions indicates that this genotype seems particularly vulnerable under more challenging environmental conditions. This reduced elongation suggests that ‘Dacke’ and similarly responding genotypes may experience greater physiological constraints when subjected to combined osmotic and cold stress. The physiological limitations could include inhibited cell division, reduced water uptake, and impaired metabolic processes necessary for root growth (Equiza and Tognetti, 2001; Hassan et al., 2021). Additionally, osmotic stress can induce oxidative stress and disrupt hormonal balance, further impairing the ability of plants to sustain root development (Hassan et al., 2021).

The enhanced root performance of ‘Diskett’ and ‘KWS Alderon’, which maintained longer root length under the drought and cold stress conditions simulated here, points to genetic differences that influence how these plants allocate resources under stress. Interestingly, the PCA results showed that after 7 days of PEG-induced osmotic stress at 10 °C, genotypes clustered more closely, suggesting a temporary homogenization in root responses, likely due to a general early-phase reduction in elongation and thickening as a protective measure to minimize water loss and conserve energy. However, the ability of certain genotypes to recover or maintain growth despite this initial convergence may reflect adaptive traits that promote sustained water uptake and root development. These genotypes may possess genetic adaptations that enable them to maintain water uptake and root growth despite the osmotic challenges imposed by PEG at 7°C. Studies have shown that genotypes with enhanced root plasticity tend to adjust their root architecture more effectively, such as through increased root branching or deeper root systems, allowing them to continue exploring the soil for moisture and nutrients under stress (Li et al., 2021). This differential response also suggests the possibility of variations in the expression of genes related to osmotic stress tolerance, such as those involved in aquaporin regulation or osmoprotectant production (Ayadi et al., 2019; Tang et al., 2023), which may allow some genotypes to cope better with dehydration and maintain root growth under low water potential conditions (Shekoofa and Sinclair, 2018; Shivaraj et al., 2021).

### 4.2 Thinner roots as an adaptive mechanism for traits related to nitrogen use efficiency

Thinner roots in some genotypes may be an adaptive strategy for coping with limited water availability. Thus, thinner roots, with more metaxylem vessels, have been shown to facilitate more efficient water uptake under drought stress (Chen et al., 2021; Liu et al., 2022b). Furthermore, our results align with the second hypothesis (H2), which suggests that prolonged exposure to combined stress conditions (low temperatures and drought) would lead to significant variation in root diameter among genotypes. Liu et al. (2022b), who investigated partly the same material as in our study, found that shallow, thin embryonic roots can enhance N uptake efficiency but may reduce N conversion efficiency. Consistent with Liu et al. (2022b), genotypes such as ‘KWS Alderon’, that excel in N conversion efficiency, typically develop fewer, thicker roots with larger metaxylem vessels, which is supported by our results. ‘Bjarne’ and ‘Quarna’ exhibited shorter root diameters compared to ‘Diskett’ and ‘Dacke’ under both control and stress conditions (PEG at 10°C-7 days and 10°C-14 days). This may reflect an adaptive phase, where some genotypes resumed elongation or maintained root thickness, indicating varied capacities to tolerate or adapt to prolonged osmotic stress. According to Liu et al. (2022b) genotypes with greater N uptake efficiency tend to exhibit shallower root angles, a higher number of thinner roots, and an increase in metaxylem vessels. This insight provides a context for interpreting the results in the same genotypes used in our study, where thinner roots may impact nutrient absorption and early vigor under prolonged drought conditions. Such findings align with the previous reports of a negative relationship, or potential trade-off, between N uptake efficiency and N conversion efficiency (Lambers and Oliveira, 2019; Weih et al., 2018), though the underlying mechanisms remain unclear. As N uptake by roots increases, leaf N concentration increments, yet photosynthetic efficiency per unit of N decreases (Lambers and Oliveira, 2019). In Liu et al. (2022b), it is suggested that this opposite relationship between N uptake efficiency and N conversion efficiency mirrors negative associations between certain root traits (i.e., root number and diameter, metaxylem number and diameter) observed in early growth stage. It has been suggested that N conversion efficiency tends to be negatively correlated with grain N concentration (Weih et al., 2018), which could indicate a trade-off between a high-yield trait (N conversion efficiency) and a quality –related trait (grain N concentration). This potential trade-off was documented by Fowler (2003) and Amiri et al. (2018) as well.

This inverse relationship between breeding targets underscores a key challenge in efforts to cultivate genotypes that combine both yield and quality attributes. In our study, root diameter displayed a negative relationship across genotypes with distinct breeding objectives. Genotypes with thinner roots were generally those bred for high protein content, as observed under control conditions and in the, 10°C-7 days, and 10°C-14 days treatments. High-protein genotypes such as ‘Bjarne’ and ‘Quarna’, showed thinner roots in three of the treatments compared to intermediate and high-yield genotypes like ‘KWS Alderon’. This aligns with the previously explained classifications by Guardia-Velarde et al. (2023).

Further, our findings corroborate Liu et al. (2022b), who demonstrated that thinner roots correlate with increased nitrogen uptake efficiency and grain nitrogen concentration, while thicker roots align with higher nitrogen conversion efficiency. Moreover, while thinner roots can often be associated with enhanced nitrogen uptake (Liu et al., 2022b), this relationship does not always necessarily equate to improved nitrogen conversion efficiency within the grain (Flynn et al., 2023; Li et al., 2024). The connection between root thickness and N uptake or grain N concentration is influenced by various factors such as drought and fertilizer placement depth (Bryant-Schlobohm et al., 2020), making it a more complex relationship rather than a straightforward correlation across all wheat genotypes. Additionally, N accumulation efficiency (U_N_) and N conversion efficiency (E_N_) are known to often exhibit a negative relationship (Maire et al., 2009; Weih et al., 2018). From Liu et al. (2022b) and our research, we can suggest that E_N_ was associated with high-yield genotypes like ‘KWS Alderon’, whereas grain N concentration, related to protein content, was linked to genotypes such as ‘Bjarne’ and ‘Quarna’. In this study, high-protein genotypes demonstrated longer roots across the control, 7°C-7 days, 10°C-7 days, and 10°C-14 days treatments compared to high-yield and intermediate genotypes, highlighting distinct root architecture patterns associated with nutrient efficiency and breeding goals under these combined stress conditions.

### 4.3 Photoperiod sensitivity regulates root development timing

The molecular analysis of wheat genotypes regarding photoperiod sensitivity (*PPD-D1* gene) and vernalization requirements (*VRN-1* genes) revealed significant genetic diversity, which may be critical in root development under varying environmental conditions.

The *PPD-D1* gene, responsible for controlling the plant’s response to photoperiod (Kiss et al., 2014), was found to vary among the genotypes, with ‘Bjarne’ exhibiting the photoperiod-insensitive allele (*Ppd-D1a*). This allele allows earlier flowering regardless of day length (Seki et al., 2011; Shcherban et al., 2014), potentially enabling ‘Bjarne’ to allocate resources to root development earlier in the growing season compared to photoperiod-sensitive genotypes like ‘Dacke’, ‘Diskett’, ‘Rohan’, ‘Quarna’ and ‘KWS Alderon’, which carry the sensitive *ppd-D1b* allele. ‘Bjarne’s’ photoperiod insensitivity likely contributes to its superior root length under stress conditions, as observed in the PEG treatments at 7°C and 10°C in our study. By initiating development earlier, ‘Bjarne’ may redirect energy resources toward developing a robust root system, thereby enhancing its ability to absorb water and nutrients under stress. This is consistent with findings by Makhoul et al. (2024), which demonstrated that genotypes carrying the *Ppd-D1a* allele exhibited deeper roots than those carrying *ppd-D1b*. Similarly, in our study, ‘Bjarne’ (*Ppd-D1a*) showed faster root development, as indicated by increased root length. In contrast, photoperiod-sensitive genotypes, which flower based on day length, might experience delayed resource allocation to root growth, limiting their ability to adapt to stress.

### 4.4 Temperature affects developmental rate and yield

The timing of heading in wheat is a critical adaptation to varying environmental stresses, particularly drought and temperature extremes. Early flowering, or early heading, can be advantageous in regions where high temperatures (Mondal et al., 2013) or drought conditions (Varga et al., 2014; Shavrukov et al., 2017) significantly reduce the length of the growing season. This drought-avoidance strategy enables plants to complete key developmental stages, such as flowering and grain filling, before the full onset of drought (Blum, 2011). However, this adaptation is only beneficial if drought stress does not overlap with these sensitive phases. In contrast, later-heading genotypes may exhibit an adaptive advantage when drought is prolonged, as the delayed development can shift critical growth stages to potentially less drought-stressed periods (Verbraeken et al., 2021; Frantová et al., 2022).

Temperature further complicates these stress responses, as it influences both wheat yield and protein content. High temperatures accelerate development and shorten the growing season (Innes et al., 2015), often resulting in reduced grain yield but increased protein content, as resources are redirected towards synthesizing proteins that bolster heat stress resilience (Ullah et al., 2022; Zhao et al., 2022). On the other hand, mild low temperatures during grain filling extend the duration of this phase, leading to higher yields and lower protein content, a response observed in a study by Uhlen et al. (2015). In their study, the selected spring wheat genotypes typically grown in Norway were also cultivated in Minnesota over three seasons and they exhibited higher yields and lower protein levels under Norwegian cooler conditions, while the warmer Minnesota climate produced shorter growing seasons, lower yields, and higher protein levels in the same genotypes, including ‘Bjarne’ and ‘Quarna’. Extreme cold, however, prompts wheat to synthesize protective proteins, which, while enhancing survival, reduces yield and increases protein concentration due to cold-induced metabolic adjustments (Ji et al., 2017; Trischuk et al., 2014; Shi et al., 2022).

Furthermore, genotypic variations, particularly the allelic composition of the *VRN-1* locus, contribute significantly to low temperature tolerance. Recessive alleles of *VRN-1* confer greater cold tolerance than spring-dominant alleles, enabling winter genotypes to withstand low temperatures during sensitive growth stages (Kobayashi et al., 2005; Dhillon et al., 2010; Cuesta-Marcos et al., 2015), thereby stabilizing yield potential in cooler environments. This genetic adaptation illustrates the role of allele-specific responses in breeding wheat genotypes with improved resilience to climate challenges.

### 4.5 Facultative growth habits revealed through vernalization pathway variation and their influence on root development

The *VRN-1* gene regulates the vernalization requirement in wheat (Loukoianov et al., 2005; Santra et al., 2009; Chen et al., 2018), and our results showed clear genotype variation across the studied plant material. The genotypes ‘Bjarne’, ‘Rohan’, and ‘KWS Alderon’ exhibited winter alleles (*vrn-A1/vrn-B1*), typically requiring cold exposure to flower. In contrast, ‘Dacke’ and ‘Diskett’ carried a combination of spring and winter alleles, indicating reduced vernalization requirements. These genetic differences may partly explain the variation in root characteristics observed under drought and cold stress, as the vernalization pathway can influence the balance between vegetative growth (including roots) and the transition to reproductive development (Yan et al., 2015; Ochagavia et al., 2022).

Mutations in *VRN-D1* were also detected, particularly in the size of PCR products for the winter allele. While a typical *vrn-D1* fragment is expected at ~990 bp, several of the here investigated genotypes showed larger products (~1200 bp), which may indicate insertions in intron 1 region. Such structural changes have been reported previously (Fu et al., 2005; Muterko et al., 2015; Yan et al., 2015; Chepurnov et al., 2023) and are known to affect gene regulation. These regulatory differences likely contribute to a facultative growth habit, allowing these genotypes to be successfully spring-sown under Swedish conditions, unlike true winter wheat, which would fail to flower without sufficient vernalization.

Although this study did not include sequencing of the *VRN-1* promoter, the analysis of intron 1 and exon 4 of *VRN-A1*, along with *VRN-B1* and *VRN-D1*, provided sufficient resolution to distinguish between winter and facultative types. The identified polymorphisms, particularly deletions and SNPs, have been shown to be reliable molecular markers (Yan et al., 2015). Based on this data, we confirm that all genotypes studied here exhibit a facultative growth habit, rather than being true spring wheat.

The genotyping results for *VRN-1* (intron 1 and exon 4) and *PPD-D1* indicated an inferred growth habit (“Habit”) for each genotype, based on reverse genetics (**Table 2**). This means the phenotype was predicted from known gene-function relationships, rather than derived from direct observation. While all genotypes were originally labeled as spring wheat, their allele combinations revealed a facultative profile, capable of adapting to both spring and winter sowing. According to Fu et al. (2005), true spring wheat carries dominant alleles at all *VRN-1* loci (*Vrn-A1, Vrn-B1, Vrn-D1*). The “Heading time*” column provides an approximate prediction based on allele composition, such as the presence of *Ppd-D1a* and spring *VRN-B1* alleles; however, these predictions remain tentative and should be validated through phenotypic evaluation under field or controlled conditions, as heading time is influenced by complex interactions between genotype and environment.

**Table 2:** Results of experimental genotyping and assumed growth habits of selected wheat genotypes in this study.

Genotype	*VRN-1* (A genome, intron 1)	*VRN-1* (B genome, intron 1)	*VRN-1* (D genome, intron 1)	*VRN-A1* (exon 4)	*PPD-D1*	Habit	Heading time*
BJARNE	*vrn-A1*	*vrn-B1*	Mutated *vrn-D1* (likely spring)	*Vrn-A1b*	*Ppd-D1a*	facultative	Earliest according to Uhlen et al., 2015
DACKE		*Vrn-B1*			*ppd-D1b*	facultative	Medium-early
DISKETT		*Vrn-B1*			*ppd-D1b*	facultative	Medium-early
ROHAN		*vrn-B1*			*ppd-D1b*	facultative	Later
QUARNA		Not determined			*ppd-D1b*	facultative	Medium-early
KWS ALDERON		*vrn-B1*			*ppd-D1b*	facultative	Later

*Approximate, according to the reverse genetics approach.

### 4.6 Genotype-specific root adaptations patterns enhance stress resilience

The observed differences in root morphology across genotypes indicated a range of adaptive strategies, supporting our third hypothesis (H3) that the correlation between root length and diameter is genotype-dependent. This variability likely reflects genotypic adaptations in response to osmotic stress, suggesting distinct patterns in how different wheat genotypes adjust their root architecture to optimize resource acquisition. These adaptive variations in phenotypic patterns may be influenced by specific alleles, such as *PPD-D1* and *VRN-1*, which play critical roles in shaping root traits (Brasier et al., 2018; Güleç et al., 2022).

For instance, genotypes with the photoperiod-insensitive *Ppd-D1a* allele, such as ‘Bjarne’, exhibit earlier root growth initiation, which could facilitate deeper or more robust root systems under stress. This genetic advantage may enable genotypes carrying *Ppd-D1a* to allocate resources more effectively to root development during early growth stages. In contrast, genotypes with the photoperiod-sensitive *ppd-D1b* allele might delay root elongation, which could affect their ability to adjust root diameter in response to stress, prioritizing reproductive growth in later stages instead.

Similarly, the *VRN-1* gene, influencing vernalization requirements, may drive differences in root morphology adaptation (Güleç et al., 2022). Genotypes with a facultative vernalization requirement might display flexible root development, responding dynamically to environmental conditions by adjusting root length and diameter to optimize resource uptake. This genetic flexibility could provide an advantage in environments where drought conditions fluctuate, allowing these genotypes to maintain root growth and resource acquisition under suboptimal temperatures and osmotic stress.

The correlation between root length and diameter observed across genotypes highlights a potential link between these morphological traits and genetic factors, underscoring the importance of selecting specific alleles in breeding programs focused on enhancing stress tolerance (Beyer et al., 2019). The differential expression of *PPD-D1* and *VRN-1* in response to environmental cues may therefore serve as markers for identifying genotypes with adaptive root traits suitable for drought-prone regions. These genetic traits should be considered in breeding programs to improve root plasticity, stress tolerance, and overall productivity in wheat. Nevertheless, more studies on larger datasets of genotypes of different origin are needed to confirm these statements fully. Other gene genotyping, such *VRN-2*, *VRN-3*, *PPD-B1*, *PPD-A1*, and *PPD-2*, needs to follow their expression profiles during root growth.

### 4.7 Advancing wheat breeding for drought resilience

Root system architecture has long been recognized as a vital factor in drought tolerance, with thin, deep roots associated with better N uptake and thicker roots correlated with increased stability and water retention (Gregory and Brown, 1989; Reynolds et al., 2007; Manschadi et al., 2006). This study’s findings reinforce the importance of selecting for root traits that enhance nutrient use efficiency under stress. High-protein genotypes, such as ‘Bjarne’ and ‘Quarna’, displayed thinner roots, suggesting that thinner root morphology may correlate with efficient N uptake, particularly under osmotic stress. This trait could be advantageous in environments where soil N is limited or water is scarce, as thinner roots may better exploit localized nutrient patches while conserving water (Liu et al., 2022b; Rossi et al., 2024). In contrast, the high-yield genotype ‘KWS Alderon’ tended to develop thicker roots under certain stress conditions, which may enhance its structural stability and help it better withstand stress when moisture is more accessible in the upper soil layers. A study by Foulkes et al. (2009) and a minireview by Chawade et al. (2018) pointed out that root thickness and architecture play essential roles in how wheat adapts to moisture conditions, supporting a targeted breeding approach that integrates yield potential with specific root traits tailored to different stress environments.

To breed wheat genotypes resilient to drought and adaptable to diverse environments, it is essential to prioritize genotypes that optimize water and nutrient acquisition. Selecting for genotypes with a combination of root length, diameter, and plasticity would support resilience under both mild and severe drought conditions, enabling wheat to better withstand the variability of climate conditions projected for the Northern Hemisphere (Chawade et al., 2018). Selecting genotypes that can perceive environmental changes and adapt by inducing a plastic response would be an effective strategy to continuing growing, even under fluctuating environmental conditions (Nicotra et al., 2010). Integrating genetic markers like the *PPD-D1a* allele, which supports early root development, could further improve the plant’s capacity to establish a robust root system before drought onset. The genotypes selected for this study, commonly grown in northern Europe, have been evaluated in central Sweden for their suitability to varying breeding objectives (Liu et al., 2021a; Guardia-Velarde et al., 2023), including high-yield and high-protein profiles. Incorporating insights from these diverse objectives can guide breeding efforts to improve root traits and drought resilience in a way that aligns with specific agronomic goals.

The inclusion of QTLs associated with root dry weight, N uptake, and root length density (Pang et al., 2014; Rossi et al., 2024) could be beneficial, especially for spring wheat adapted to high latitudes. Increased root length density, for instance, is linked to greater below-ground resource capture, supporting drought tolerance and nutrient use efficiency under stressful conditions (Foulkes et al., 2009). Identifying and incorporating QTLs like *QMrl-7B*, which is associated with a robust root system and high N use efficiency, could enhance drought resilience by promoting root growth and water uptake even during early development stages (Rossi et al., 2024).

## 5 Conclusion

Our findings indicate that specific root traits, such as length and diameter, significantly influence a genotype’s adaptability to drought, with variations observed both in optimal conditions and under simulated stress conditions. Genotypes like ‘Bjarne’ and ‘KWS Alderon’ demonstrated contrasting responses to the simulated stress conditions, with thinner, elongated roots for deeper water access and thicker roots for stability, respectively, suggesting that targeted root traits may confer advantages in water-limited environments. The genetic analysis revealed that photoperiod sensitivity and vernalization requirements seem to be associated with root architecture plasticity, with photoperiod-insensitive and facultative genotypes showing early root development and potential resilience under fluctuating moisture availability. These insights emphasize the value of integrating both root traits and genes or genetic markers into breeding programs focused on enhancing drought tolerance. In conclusion, this study underscores the potential of breeding wheat genotypes with optimized root architectures and genetic flexibility for improved drought resilience.

## Data Availability

The original contributions presented in the study are included in the article/**Supplementary Material**. Further inquiries can be directed to the corresponding author.
