# Supplementary material for: Genotype adaptive patterns in spring wheat reveal drought-induced differentiation in root morphology
Source: Front Plant Sci. 2025 May 21;16:1534211. doi: 10.3389/fpls.2025.1534211 (PMC12134626; doi:10.3389/fpls.2025.1534211)
Supplement: Supplementary file 1 [file Table1.docx]

Supplementary Material

# Weather conditions and field management for maternal plants during growing season 2021

The 2021 growing season in Uppsala, Sweden, was characterized by weather variations (Supplementary Figure 1). May and July showed particularly pronounced temperature increases, typical for this period, with mean air temperatures reaching 10.1°C and 20.3°C, respectively. These elevated temperatures, especially during July, were coupled with significantly lower-than-average precipitation (2018-2023), particularly in July, which only received 14.8 mm of rainfall. The combination of high temperatures and low precipitation likely led to pronounced drought stress during critical growth stages, influencing plant development, yield, and the quality of seeds used in subsequent root vigor testing. While the precipitation in May was relatively high at 86.6 mm, providing some early-season moisture, the cumulative stress from drier conditions in July and September would have had lasting effects on the crops. Despite any late-season relief from increased rainfall in August, the overall challenging weather conditions likely imposed significant stress on the plants, affecting their growth and development throughout the season.


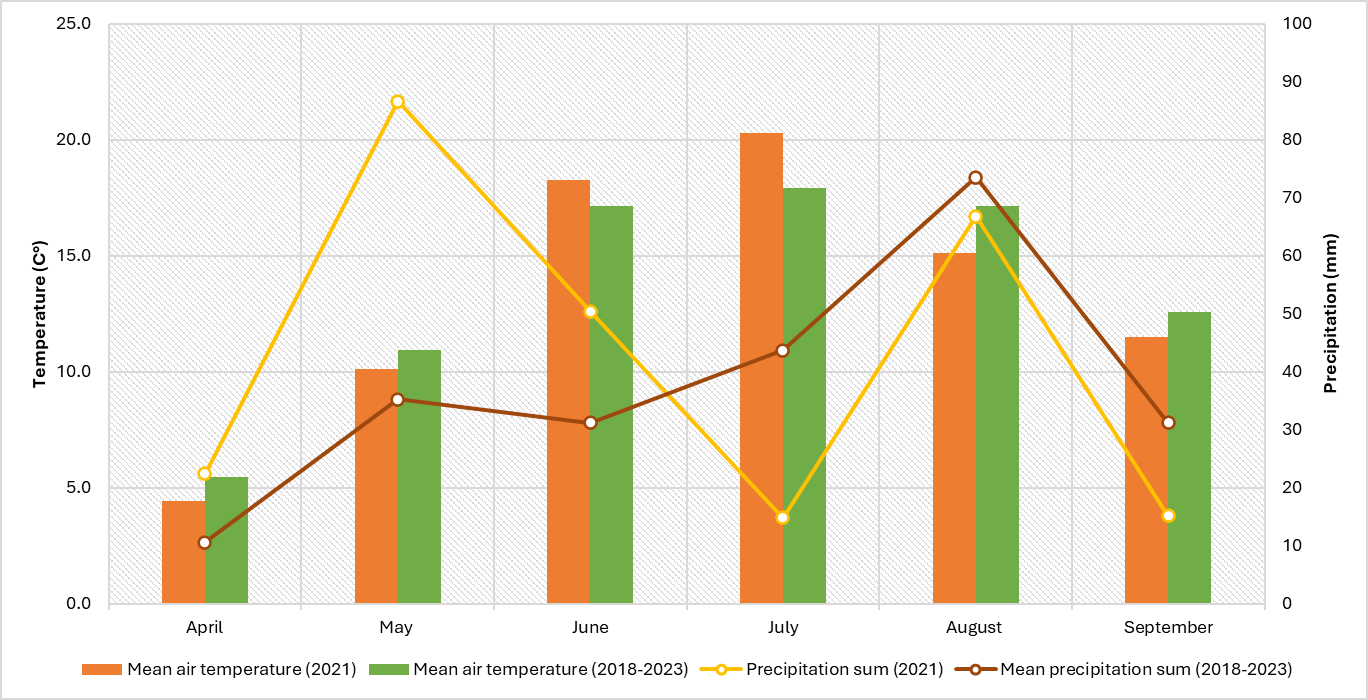


**Supplementary Figure 1.**  The weather conditions in 2021 compared to 6 year averages in mean temperature and precipitation

# Detailed protocol for allelic detection of *PPD-D1* and *VRN-1*

Three leaves were collected from the seedlings of each genotype, grown at room temperature in a soil substrate, for DNA extraction using the DNeasy Plant Mini Kit (Qiagen, Germany) according to the manufacturer's protocol.

**For PCR amplification**

*PPD-D1* alleles were detected using primers from Beales et al. (2007), yielding 288 bp (dominant, insensitive) and 414 bp (recessive, sensitive) products.

*VRN-1* genes (*VRN-A1, VRN-B1, VRN-D1*) were amplified using primers from Fu et al. (2005) to identify large deletions in **intron 1**:

*VRN-A1*: 1068 bp (winter allele)

*VRN-B1*: 709 bp (spring allele), 1149 bp (winter allele)

*VRN-D1*: 997 bp (winter allele)

Additionally, a region covering **exon 4** of *VRN-A1* was amplified to detect SNPs associated with reduced vernalization requirements (Yan et al., 2015). PCR products were digested with SphI and resolved on 2% agarose gels, showing polymorphic bands:

vrn-A1a: 199 bp (facultative, ~3 weeks vernalization)

vrn-A1b: 221 bp (winter, ~6 weeks vernalization)

Reference genotypes Tybalt (facultative) and Bohemia (strict winter) were included.

PCR was carried out using the Taq DNA Polymerase Kit (Qiagen, Germany). Reaction volumes were 20 µL. For VRN-1, MgCl₂ was adjusted to 2 mM and Q-solution was added in equal volume to the coral buffer to enhance amplification specificity. PCR products were separated on agarose gels:

1.5% agarose gel for *PPD-D1*

1% agarose gel for *VRN-1*

2% agarose gel for *Vrn-A1* (exon 4)

Gels were run in TBE buffer, and a 100 bp DNA ladder (Elisabeth Pharmacon, Czech Republic; 100–1517 bp range) was used as the molecular weight standard. Gel images were cropped for clarity, a common practice when comparing PCR products of different sizes.

# Spearman correlation statistical significance and r values

**Supplementary Table 1.** The r values of Spearman correlation and statistical significance

| Treatment | Genotype | Spearman correlation | p-value |
| --- | --- | --- | --- |
| Control | Bjarne | 0.203 | 0.527 |
|  | Dacke | 0.629 | 0.028 |
|  | Diskett | 0.049 | 0.88 |
|  | KWS Alderon | -0.713 | 0.009 |
|  | Quarna | -0.587 | 0.045 |
|  | Rohan | 0.252 | 0.43 |
| PEG 10 degree, 7 days | Bjarne | 0.007 | 0.983 |
|  | Dacke | -0.056 | 0.863 |
|  | Diskett | -0.545 | 0.067 |
|  | KWS Alderon | -0.336 | 0.286 |
|  | Quarna | -0.084 | 0.795 |
|  | Rohan | -0.329 | 0.297 |
| PEG 10 degree, 14 days | Bjarne | -0.51 | 0.09 |
|  | Dacke | -0.587 | 0.045 |
|  | Diskett | -0.564 | 0.056 |
|  | KWS Alderon | -0.343 | 0.276 |
|  | Quarna | -0.531 | 0.075 |
|  | Rohan | -0.322 | 0.308 |
| PEG 7 degree, 7 days | Bjarne | 0.328 | 0.457 |
|  | Dacke | 0.112 | 0.729 |
|  | Diskett | -0.091 | 0.779 |
|  | KWS Alderon | -0.497 | 0.101 |
|  | Quarna | 0.259 | 0.417 |
|  | Rohan | 0.091 | 0.79 |
| PEG 7 degree, 14 days | Bjarne | -0.566 | 0.055 |
|  | Dacke | 0.105 | 0.746 |
|  | Diskett | 0.238 | 0.457 |
|  | KWS Alderon | 0.315 | 0.319 |
|  | Quarna | -0.245 | 0.442 |
|  | Rohan | 0.277 | 0.384 |

# Non-parametric testing by Kruskal-Wallis test and Dwass-Steel-Critchlow-Fligner pairwise comparison as post-hoc testing

**Supplementary Table 2.** Statistical testing of length and diameter differences among genotypes under **control treatment**

| Kruskal-Wallis | **χ^2^** | **df** | **p** |
| --- | --- | --- | --- |
| Length | 26.8 | 5 | < 0.001 |
| Diameter | 46.8 | 5 | < 0.001 |
| Pairwise comparisons | **Length** | **W** | **p** |
| Bjarne | Dacke | -3.7559 | 0.084 |
| Bjarne | Diskett | -4.3274 | 0.027 |
| Bjarne | KWS Alderon | -5.3072 | 0.002 |
| Bjarne | Qaurna | -4.1641 | 0.038 |
| Bjarne | Rohan | -4.6540 | 0.013 |
| Dacke | Diskett | -0.7348 | 0.995 |
| Dacke | KWS Alderon | -3.9192 | 0.062 |
| Dacke | Quarna | -0.2449 | 1.000 |
| Dacke | Rohan | -2.5311 | 0.473 |
| Diskett | KWS Alderon | -3.4293 | 0.148 |
| Diskett | Quarna | 1.0614 | 0.976 |
| Diskett | Rohan | -1.6330 | 0.858 |
| KWS Alderon | Quarna | 4.4091 | 0.023 |
| KWS Alderon | Rohan | 0.0816 | 1.000 |
| Quarna | Rohan | -2.4495 | 0.511 |
| Pairwise comparisons | **Diameter** | **W** | **p** |
| Bjarne | Dacke | 5.879 | < 0.001 |
| Bjarne | Diskett | 5.144 | 0.004 |
| Bjarne | KWS Alderon | 5.879 | < 0.001 |
| Bjarne | Qaurna | 4.001 | 0.053 |
| Bjarne | Rohan | 3.674 | 0.098 |
| Dacke | Diskett | -4.981 | 0.006 |
| Dacke | KWS Alderon | 1.715 | 0.831 |
| Dacke | Quarna | -5.471 | 0.002 |
| Dacke | Rohan | -4.572 | 0.016 |
| Diskett | KWS Alderon | 5.307 | 0.002 |
| Diskett | Quarna | -1.796 | 0.802 |
| Diskett | Rohan | -0.694 | 0.997 |
| KWS Alderon | Quarna | -5.471 | 0.002 |
| KWS Alderon | Rohan | -4.817 | 0.009 |
| Quarna | Rohan | 0.653 | 0.997 |

**Supplementary Table 3.** Statistical testing of length and diameter differences among genotypes under **PEG 7 degree and 7 day-treatment**

| Kruskal-Wallis | **χ^2^** | **df** | **p** |
| --- | --- | --- | --- |
| Length | 17.80 | 5 | 0.003 |
| Diameter | 4.91 | 5 | 0.427 |
| Pairwise comparisons | **Length** | **W** | **p** |
| Bjarne | Dacke | 2.8577 | 0.330 |
| Bjarne | Diskett | -2.5311 | 0.473 |
| Bjarne | KWS Alderon | -2.5311 | 0.473 |
| Bjarne | Qaurna | -1.7963 | 0.802 |
| Bjarne | Rohan | -2.2630 | 0.599 |
| Dacke | Diskett | -4.0825 | 0.045 |
| Dacke | KWS Alderon | -4.5724 | 0.016 |
| Dacke | Quarna | -3.1843 | 0.215 |
| Dacke | Rohan | -4.7871 | 0.009 |
| Diskett | KWS Alderon | -1.3064 | 0.941 |
| Diskett | Quarna | 0.4899 | 0.999 |
| Diskett | Rohan | 0.0870 | 1.000 |
| KWS Alderon | Quarna | 1.7963 | 0.802 |
| KWS Alderon | Rohan | 0.6963 | 0.996 |
| Quarna | Rohan | -0.4352 | 1.000 |

**Supplementary Table 4.** Statistical testing of length and diameter differences among genotypes under **PEG 7 degree and 14 day-treatment**

| Kruskal-Wallis | **χ^2^** | **df** | **p** |
| --- | --- | --- | --- |
| Length | 7.59 | 5 | 0.180 |
| Diameter | 28.49 | 5 | < 0.001 |
| Pairwise comparisons | **Diameter** | **W** | **p** |
| Bjarne | Dacke | 0.898 | 0.988 |
| Bjarne | Diskett | -0.490 | 0.999 |
| Bjarne | KWS Alderon | -0.163 | 1.000 |
| Bjarne | Qaurna | 1.225 | 0.955 |
| Bjarne | Rohan | -2.041 | 0.701 |
| Dacke | Diskett | -2.531 | 0.473 |
| Dacke | KWS Alderon | -1.960 | 0.736 |
| Dacke | Quarna | 0.245 | 1.000 |
| Dacke | Rohan | -3.348 | 0.168 |
| Diskett | KWS Alderon | 0.735 | 0.995 |
| Diskett | Quarna | 1.633 | 0.858 |
| Diskett | Rohan | -1.225 | 0.955 |
| KWS Alderon | Quarna | 1.388 | 0.924 |
| KWS Alderon | Rohan | -1.960 | 0.736 |
| Quarna | Rohan | -2.613 | 0.435 |

**Supplementary Table 5.** Statistical testing of length and diameter differences among genotypes under **PEG 10 degree and 7 day-treatment**

| Kruskal-Wallis | **χ^2^** | **df** | **p** |
| --- | --- | --- | --- |
| Length | 16.5 | 5 | 0.005 |
| Diameter | 40.5 | 5 | < 0.001 |
| Pairwise comparisons | **Length** | **W** | **p** |
| Bjarne | Dacke | 1.7963 | 0.802 |
| Bjarne | Diskett | -1.6330 | 0.858 |
| Bjarne | KWS Alderon | -1.3064 | 0.941 |
| Bjarne | Qaurna | 0.0816 | 1.000 |
| Bjarne | Rohan | -2.5311 | 0.473 |
| Dacke | Diskett | -4.2458 | 0.032 |
| Dacke | KWS Alderon | -3.7559 | 0.084 |
| Dacke | Quarna | -2.0412 | 0.701 |
| Dacke | Rohan | -4.8990 | 0.007 |
| Diskett | KWS Alderon | 0.3266 | 1.000 |
| Diskett | Quarna | 2.2045 | 0.626 |
| Diskett | Rohan | -1.3064 | 0.941 |
| KWS Alderon | Quarna | 1.3064 | 0.941 |
| KWS Alderon | Rohan | -1.7146 | 0.831 |
| Quarna | Rohan | -3.4293 | 0.148 |
| Pairwise comparisons | **Diameter** | **W** | **p** |
| Bjarne | Dacke | 5.840 | < 0.001 |
| Bjarne | Diskett | 4.328 | 0.027 |
| Bjarne | KWS Alderon | 5.677 | < 0.001 |
| Bjarne | Qaurna | 1.960 | 0.736 |
| Bjarne | Rohan | 4.737 | 0.011 |
| Dacke | Diskett | -4.409 | 0.023 |
| Dacke | KWS Alderon | -1.021 | 0.979 |
| Dacke | Quarna | -5.797 | < 0.001 |
| Dacke | Rohan | -3.184 | 0.215 |
| Diskett | KWS Alderon | 3.471 | 0.138 |
| Diskett | Quarna | -2.858 | 0.330 |
| Diskett | Rohan | 0.735 | 0.995 |
| KWS Alderon | Quarna | -5.226 | 0.003 |
| KWS Alderon | Rohan | -2.694 | 0.399 |
| Quarna | Rohan | 3.348 | 0.168 |

**Supplementary Table 6.** Statistical testing of length and diameter differences among genotypes under **PEG 10 degree and 14 day-treatment**

| Kruskal-Wallis | **χ^2^** | **df** | **p** |
| --- | --- | --- | --- |
| Length | 15.7 | 5 | 0.008 |
| Diameter | 33.9 | 5 | < 0.001 |
| Pairwise comparisons | **Length** | **W** | **p** |
| Bjarne | Dacke | 0.6532 | 0.997 |
| Bjarne | Diskett | 0.4899 | 0.999 |
| Bjarne | KWS Alderon | -0.4082 | 1.000 |
| Bjarne | Qaurna | 1.7146 | 0.831 |
| Bjarne | Rohan | -2.1229 | 0.664 |
| Dacke | Diskett | -0.0816 | 1.000 |
| Dacke | KWS Alderon | -2.5311 | 0.473 |
| Dacke | Quarna | 1.8779 | 0.770 |
| Dacke | Rohan | -4.0825 | 0.045 |
| Diskett | KWS Alderon | -1.6330 | 0.858 |
| Diskett | Quarna | 2.2045 | 0.626 |
| Diskett | Rohan | -3.5926 | 0.113 |
| KWS Alderon | Quarna | 3.3476 | 0.168 |
| KWS Alderon | Rohan | -2.5311 | 0.473 |
| Quarna | Rohan | -4.6540 | 0.013 |
| Pairwise comparisons | **Diameter** | **W** | **p** |
| Bjarne | Dacke | 4.7357 | 0.011 |
| Bjarne | Diskett | 3.9200 | 0.062 |
| Bjarne | KWS Alderon | 5.8788 | < 0.001 |
| Bjarne | Qaurna | 1.4697 | 0.905 |
| Bjarne | Rohan | 4.9806 | 0.006 |
| Dacke | Diskett | -1.5517 | 0.883 |
| Dacke | KWS Alderon | 4.5724 | 0.016 |
| Dacke | Quarna | -2.9394 | 0.299 |
| Dacke | Rohan | 0.0816 | 1.000 |
| Diskett | KWS Alderon | 4.0017 | 0.053 |
| Diskett | Quarna | -2.7785 | 0.363 |
| Diskett | Rohan | 2.1234 | 0.664 |
| KWS Alderon | Quarna | -4.7357 | 0.011 |
| KWS Alderon | Rohan | -3.4293 | 0.148 |
| Quarna | Rohan | 3.6742 | 0.098 |

# Higher resolution of y-axis for the values of root length under PEG 7 degree 7-day treatment

As the chart with all treatments and genotypes values may hide some differences under PEG 7 7-day treatment, here is a separated chart for better representation (Supplementary Figure 2).


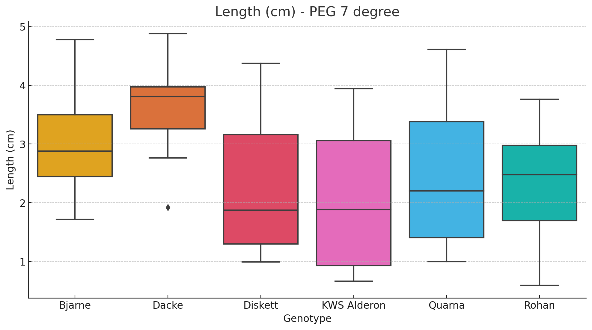


**Supplementary Figure 2**. Box plots representing the root length value under PEG 7 degree 7-day treatment
